# Supplementary material for: Targeted long-read cDNA sequencing reveals novel splice-altering pathogenic variants causing retinal dystrophies
Source: HGG Adv. 2025 Apr 18;6(3):100442. doi: 10.1016/j.xhgg.2025.100442 (PMC12099450; doi:10.1016/j.xhgg.2025.100442)
Supplement: Document S1. Figures S1 and S2 and Table S3 [file mmc1.pdf]

**HGGA, Volume *6***

## Supplemental information

# Targeted long-read cDNA sequencing reveals novel splice-altering pathogenic variants causing retinal dystrophies

**Dalila Capasso, Roberta Zeuli, Gavin Arno, Michael Kwint, Raoul Timmermans, Karla A. Ruiz-Ceja, Marianthi Karali, Francesca Simonelli, Sabrina Signorini, Enza Maria Valente, Frans P.M. Cremers, Sandro Banfi, Susanne Roosing, Daan M. Panneman, and Suzanne E. de Bruijn**

## TABLE OF CONTENTS

**SUPPLEMENTAL FIGURES……………………………………………………………………………………………………………2**

Figure S1……………………………………………………………………………………………………………………………………2

Figure S2……………………………………………………………………………………………………………………………………3

**SUPPLEMENTAL TABLES……………………………………………………………………………………………………………4**

Table S1 (Large Excel file, uploaded separately)

Table S2 (Large Excel file, uploaded separately)

Table S3……………………………………………………………………………………………………………………………………4

**SUPPLEMENTAL REFERENCES………………………………………………………………………………………………5**

## SUPPLEMENTAL FIGURES

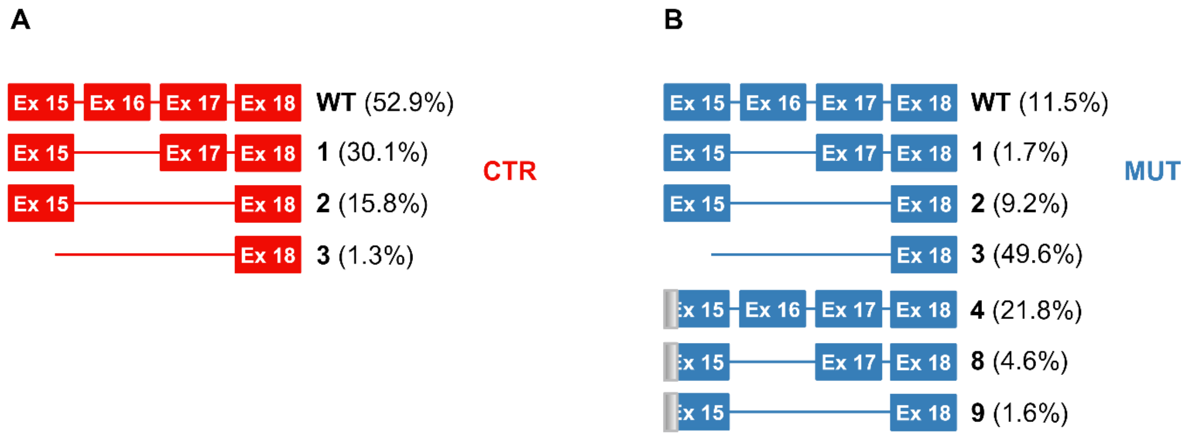


**Figure S1. Schematic representation of the midigene assay results described by Vázquez-Domínguez et al. studying the *IMPG2* c.[3023-15T>A;3023G>A] variant.** A midigene assay using wildtype and mutant midigene constructs containing exons 15-18 of *IMPG2* was previously performed. The different isoforms identified in these experiments are schematically illustrated. (A) Midigene results obtained using the wildtype *IMPG2* construct (CTR, in red). Besides the normally spliced *IMPG2* transcript (wildtype (WT)), also isoforms lacking exon 16 (isoform 1), exons 16 and 17 (isoform 2) and exons 15, 16 and 17 (isoform 3) were identified. (B) Midigene results obtained using the mutated *IMPG2* construct (MUT, in blue). Besides the wildtype isoform and isoforms 1-3, also mutant-specific isoforms were detected that included a truncation of exon 15 (isoform 4) with or without co-skipping of exon 16 (isoform 8) or exons 16 and 17 (isoform 9). Percentages indicate the relative abundance of each isoform as calculated using a semi-quantitative analysis of the RT-PCR results as described by Vázquez-Domínguez et al. [1]. Ex, Exon.

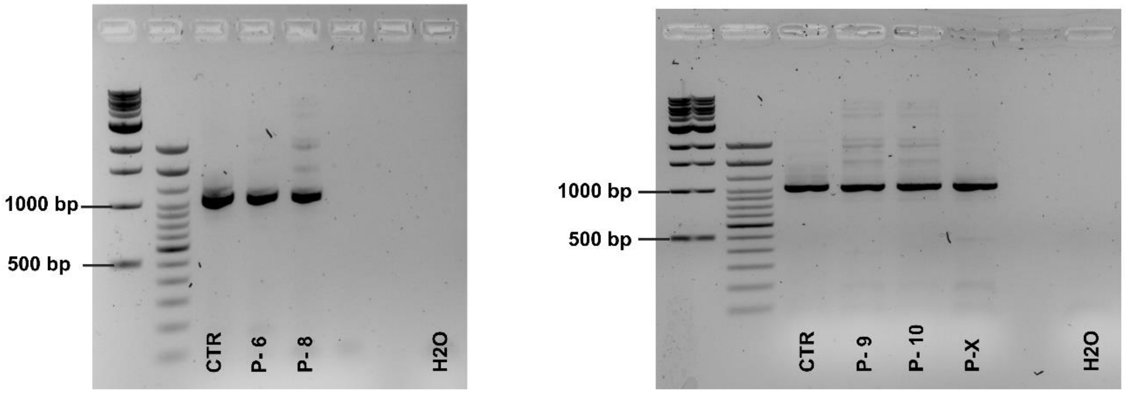


**Figure S2. Targeted long-read cDNA sequencing identifies an SVA_F insertion in *NMNAT1*.** Gel electrophoresis analysis of RT-PCR products. The complete *NMNAT1* transcript (exon 1 – exon 5) was amplified using P-6-, P-8-, P-9- and P-10-derived cDNA. Besides the expected wildtype product (993 bp), products of unexpected sizes were observed in all three affected monoallelic individuals. Bp, Base pairs; CTR, Control; P, Proband.

## SUPPLEMENTAL TABLES

**Table S3. Primer sequences and PCR conditions used.**

| Transcript | Primer F (5'-3') | Primer R (5'-3') | Length | PCR conditions |
|---|---|---|---|---|
| ***HGSNAT*** | GATGGATCAGGCTTTGCTAC | GTGGGACTGGTTGTCCTTC | 1666 bp | Q5, 63C, 2 min |
| ***IMPG2*** | GCTGAATCTGCGTCTCTAAC | TCCATCTTCTCCAGGCTTC | 1690 bp | Q5, 63C, 2 min |
| ***CHM*** | TCAAGATGGCGGATACTCTC | AAGTCTCCGAGTTAGCCTCTG | 1920 bp | Q5, 63C, 2 min |
| ***CEP290*** | CTAGAAACACGGGCAACTTG | CTTTCAGCTCCACTTTGGTC | 2717 bp | Q5, 64C, 2 min |
| ***NMNAT1*** | GATCTCCGGTAGCACTCGG | CCCAGATTGTTTCAGATCCCCA | 993 bp | Q5, 68C, 2 min |

Primer sequences used for RT-PCR analysis and targeted long-read cDNA sequencing. F, Forward primer sequence; R, Reverse primer sequence; Length, Size of amplified PCR product in base pairs (bp); PCR conditions, PCR conditions employed for each specific PCR reaction specifying (1) the polymerase used, (2) the annealing temperature and (3) the elongation time applied in minutes.

## SUPPLEMENTAL REFERENCES

1. Vázquez-Domínguez, I., et al., *Identification of a Complex Allele in IMPG2 as a Cause of Adult-Onset Vitelliform Macular Dystrophy.* Investigative Ophthalmology & Visual Science, 2022. **63**(5).
