# Supplementary material for: Targeted long-read cDNA sequencing reveals novel splice-altering pathogenic variants causing retinal dystrophies
Source: HGG Adv. 2025 Apr 18;6(3):100442. doi: 10.1016/j.xhgg.2025.100442 (PMC12099450; doi:10.1016/j.xhgg.2025.100442)
Supplement: Document S2. Article plus supplemental information [file mmc4.pdf]

HGG Advances

ARTICLE

Targeted long-read cDNA sequencing reveals novel splice-altering pathogenic variants causing retinal dystrophies

Dalila Capasso,[1,2] Roberta Zeuli,[3] Gavin Arno,[4,11] Michael Kwint,[5] Raoul Timmermans,[5] Karla A. Ruiz-Ceja,[1] Marianthi Karali,[3,6] Francesca Simonelli,[6] Sabrina Signorini,[7,8] Enza Maria Valente,[9,10] Frans P.M. Cremers,[5] Sandro Banfi,[1,3,12] Susanne Roosing,[5,12] Daan M. Panneman,[5,12] and Suzanne E. de Bruijn[5,12,13,*]

Summary

Splice-altering variants are suggested to be responsible for part of the missing heritability of inherited retinal diseases (IRDs). The interpretation of these variants is challenging as functional evidence is required to validate pathogenicity. We explored the diagnostic value of a targeted long-read cDNA sequencing (lrcDNA-seq) approach to investigate IRD-associated splicing defects. For each affected individual, RNA was isolated from blood, and for each candidate gene, cDNA amplicons, spanning the complete open reading frame or multiple exons, were generated and subjected to long-read sequencing. We validated our approach by assessing previously described pathogenic splice-altering variants in IRD-associated genes. Next, we investigated six genetically unexplained affected individuals, each carrying pathogenic variant(s) in *NMNAT1*. In two probands, we provided functional validation for previously identified variants of uncertain significance present on the second allele. In four other subjects, lrcDNA-seq revealed the partial inclusion of an SVA_F retrotransposon in the *NMNAT1* mRNA, predicted to introduce a premature stop codon. We showed that targeted lrcDNA-seq is effective in characterizing splice defects and in identifying novel splice-altering variants and uncovered the IRD genetic basis for six previously unexplained subjects. We believe that the implementation of this technique has the potential to contribute to an increased diagnostic rate of IRDs.

Introduction

Inherited retinal diseases (IRDs) are a group of rare, monogenic disorders affecting photoreceptor cells that lead to vision impairment.[1] They display a high genetic heterogeneity with 311 IRD-associated genes identified to date (RetNet, http://sph.uth.edu/retnet/). The genetic heterogeneity of IRDs hampers subjects' clinical management and complicates genetic counseling, since 40% of affected individuals still lack a conclusive genetic diagnosis.[1,2]

It is estimated that 15% of IRD-associated variants cause disease by affecting pre-mRNA splicing, a process by which introns are removed from the pre-mRNA to produce a mature mRNA transcript.[3] To ensure efficient splicing, the spliceosome needs to recognize specific nucleotide motifs that are present at the intron-exon boundaries (splice sites). Splice-altering variants can be located in exonic or intronic regions and disrupt or create these splicing motifs.[3] They can prevent the spliceosome from binding the pre-mRNA and recognize existing splice sites, or they can activate weaker, cryptic sites that are otherwise unused or only used to a lesser degree.[4,5] Consequently, these variants can result in the generation of aberrant transcripts and ultimately lead to the production of a truncated protein or aberrant mRNAs that are degraded by nonsense-mediated decay (NMD).[4]

The detection and prediction of splice-altering variants has significantly improved due to the introduction of genome sequencing in combination with effective *in silico* tools (e.g., SpliceAI[6]), which allow for the interrogation of cryptic and existing splice sites. Even though these predictions assist in prioritizing potentially causative variants, functional assays are required to confirm pathogenicity and establish a conclusive genetic diagnosis.

The most efficient approach to resolve events leading to mis-splicing would be RNA sequencing (RNA-seq) in the affected tissue. However, for IRDs specifically, this approach is not feasible due to the inaccessibility of retina-derived materials of affected individuals.[6] Midigene-based splice assays have been extensively employed

[1]Telethon Institute of Genetics and Medicine, Pozzuoli, Italy; [2]Scuola Superiore Meridionale (SSM, School of Advanced Studies), Genomic and Experimental Medicine Program, Naples, Italy; [3]Department of Precision Medicine, University of Campania 'Luigi Vanvitelli', Naples, Italy; [4]NIHR Biomedical Research Centre, Moorfields Eye Hospital NHS Foundation Trust, London, UK; [5]Department of Human Genetics, Radboud University Medical Center, Nijmegen, the Netherlands; [6]Eye Clinic, Multidisciplinary Department of Medical, Surgical and Dental Sciences, University of Campania 'Luigi Vanvitelli', Naples, Italy; [7]Developmental Neuro-ophthalmology Unit, IRCCS Mondino Foundation, Pavia, Italy; [8]Child Neuropsychiatry Unit, IRCCS Mondino Foundation, Pavia, Italy; [9]Department of Molecular Medicine, University of Pavia, Pavia, Italy; [10]Neurogenetics Research Unit, IRCCS Mondino Foundation, Pavia, Italy; [11]Institute of Ophthalmology, University College London, London, UK
[12]These authors contributed equally
[13]Lead contact
*Correspondence: suzanne.debruijn@radboudumc.nl
https://doi.org/10.1016/j.xhgg.2025.100442.
© 2025 The Author(s). Published by Elsevier Inc. on behalf of American Society of Human Genetics.
This is an open access article under the CC BY license (http://creativecommons.org/licenses/by/4.0/).

as an alternative functional method.[7–9] Despite several successful applications, they still entail several limitations such as limited genomic context, potential artifacts, and the requirement of a variant-specific design and analysis.[9,10] These drawbacks have hindered its application in routine diagnostics, and an alternative approach is warranted.

In recent years, enormous progress has been made in the field of RNA studies: the introduction of long-read platforms such as Oxford Nanopore Technologies (ONT) and Pacific Biosciences has revolutionized the field as they allow the detection of complete transcript isoforms without the need to perform any bioinformatic assembly. This advantage significantly improves the recognition and unambiguous mapping of transcripts, as well as the detection of potential splicing defects, when compared to other techniques such as Sanger sequencing.[11]

Recent studies demonstrated the successful application of the ONT platform to perform targeted long-read deep sequencing using blood- or nasal epithelial cell-derived RNA to effectively elucidate the splicing defects of IRD-related genes.[11,12] Despite the poor expression of IRD-associated transcripts in readily accessible tissues such as blood, the combination of PCR amplification of the transcript of interest with targeted long-read deep sequencing could overcome this limitation.[11,12] This suggests that blood-derived RNA might be suitable to functionally investigate candidate splice variants for IRDs using a long-read sequencing approach.

In this study, we aimed at further exploring the diagnostic value of targeted long-read cDNA sequencing (lrcDNA-seq) for IRDs, first, by analyzing a subset of previously characterized splice-affecting variants (validation cohort) and, second, by extending the analysis to currently unsolved IRD-affected individuals (discovery cohort).

Material and methods

Study cohort

IRD-affected individuals selected for this study were previously subjected to clinical evaluation at the Radboud University Medical Center (Radboudumc; Nijmegen, the Netherlands), the Center for Inherited Retinal Dystrophies of the Eye Clinic, University of Campania 'Luigi Vanvitelli' (Naples, Italy), or the University of Pavia (Pavia, Italy). Probands (P-1 to P-5) harboring a known splice-altering variant, previously validated using a midigene-based splice assay or other RNA-based studies, were enrolled in the validation cohort. Probands (P-6 to P-10) harboring a mono-allelic defect in an IRD-associated gene (i.e., a heterozygous pathogenic variant with no second pathogenic variant that could be identified after applying routine diagnostics such as gene panel, exome, or genome sequencing) were enrolled in the discovery cohort. Details about all individuals enrolled in this study can be found in Table S1 (validation cohort) and Table S2 (discovery cohort). All variants in this study were classified using the American College of Medical Genetics and Genomics (ACMG)-Association for Molecular Pathology (AMP) classification system and with the aid of the Franklin Genoox platform (https://franklin.genoox.com).[13]

RNA extraction

Blood samples and blood-derived RNAs were collected from selected affected individuals using different protocols, depending on the reference center. For the samples collected at Radboudumc, blood was collected in either PAXgene Blood RNA tubes (BD Biosciences, Franklin Lakes, NJ) (P-4) or in BD Vacutainer heparin tubes (Becton Dickinson, Vianen, the Netherlands) (P-1 and P-5), followed by immortalization of B cell lymphocytes through Epstein-Barr virus (EBV) infection. RNA was then isolated using the PAXgene Blood RNA kit (Qiagen, Hilden, Germany) or using the NucleoSpin RNA Clean-up Kit (Macherey-Nagel, Düren, Germany) for the EBV-transformed blood cells, according to the manufacturer's protocols. For the affected individuals enrolled at the Eye Clinic of the University of Campania (P-2, P-3, P-6, P-8, P-9, and P-10) and the subject enrolled at the University of Pavia (P-7), blood was collected in Tempus Blood RNA Tubes (Applied Biosystems, Waltham, MA). RNA was extracted with the Tempus Spin RNA Isolation Kit (Invitrogen, Waltham, MA) according to the manufacturer's protocol. For all samples, RNA quantification was performed by DeNovix (Wilmington, DE) or NanoDrop (Thermo Fisher Scientific, Waltham, MA) and RNA quality and integrity (score >7) was checked by TapeStation analysis.

cDNA synthesis and RT-PCR

All samples were collected and underwent cDNA synthesis using SuperScript IV Reverse Transcriptase (Invitrogen) and random hexamers following an adjusted protocol to enrich for long cDNA molecules. Specifically, input RNA (500 ng) was incubated at 60°C for 10 min to allow for the linearization of long molecules. Additionally, an extended cDNA incubation was performed at 55°C for 50 min instead of the recommended 10 min.

A total of 1 μL undiluted cDNA per sample was used as input for the reverse transcription-polymerase chain reaction (RT-PCR) performed with the Q5 High-Fidelity DNA Polymerase (New England Biolabs, Ipswich, MA) or LongAmp Taq 2X Master Mix (New England Biolabs) at standard PCR conditions. Primers and PCR conditions used for each transcript are summarized in Table S3. In general, for longer cDNA molecules (>2 kb), PCRs performed using the LongAmp Taq 2X Master Mix (New England Biolabs) were found to be more efficient (data not shown). Primers were designed using the online tool OligoCalc[14] and were initially intended to amplify the entire transcripts of interest, including the untranslated regions (UTRs). For most of the transcripts, this strategy yielded a limited amplification success rate, and therefore they were subsequently located in the first and last coding exons based on the Matched Annotation from the NCBI and European Molecular Biology Laboratory-European Bioinformatics Institute (more commonly, MANE) transcript of genes of interest.

Targeted lrcDNA-seq

Following size confirmation by gel electrophoresis, PCR products were quantified using Qubit (Thermo Fisher Scientific), and an equimolar pooling of amplicons (up to three) was performed. Subsequently, a total of 500 ng PCR product was used for library preparation and subjected to targeted lrcDNA-seq using a Sequel I sequencer (Pacific Biosciences [PacBio], Menlo Park, CA).

Amplicon libraries were prepared using the SMRTbell prep kit 3.0 (PacBio), and barcodes were ligated using the corresponding barcoded adapter plate 3.0. Sequence Primer and sequencing polymerase were annealed with the use of the Sequel II Binding Kit 3.1 (PacBio). Amplicons were sequenced for 10 h with an on-plate loading concentration of 90 pM on the Sequel I platform.

After sequencing, subreads were demultiplexed using lima version 2.5.0 and combined to create a consensus sequence using CCS version 6.3.0. These reads were subsequently filtered for RQ 0.99 to obtain HiFi reads. The HiFi reads were mapped along the GRCh38 reference genome using pbmm2 version 1.8.0 with the "preset ISOSEQ" parameter. With this parameter a transcript alignment file was obtained including exon-junction information.

Sequencing results were analyzed using the Integrative Genomics Viewer (IGV) tool.[15] The percentage of altered transcripts was calculated using the number of reads spanning specific exon-exon junctions, according to the formula: (number of reads covering a specific exon-exon junction/total number of reads) × 100%.

Long-read genome sequencing

For P-9, genomic DNA was isolated from whole blood using the FlexiGene DNA Kit (Qiagen) according to the manufacturer's protocol. DNA quality and integrity (score >9) was first checked by TapeStation analysis. Long-read genome sequencing and subsequent variant calling were performed as previously described.[16] In short, a total of 7 μg genomic DNA was used to prepare a library with the SMRTbell Prep kit 3.0 (PacBio). Size selection was then performed using the BluePippin system. Primers and polymerase were annealed to the SMRTbell library using the Sequel II binding kit 3.2 (PacBio), and subsequently the library was loaded on an 8M SMRTcell. The sequencing (30 h) was performed on the Sequel IIe system using a single flow cell. The SMRT Link version 8.0.0 software was then used to generate HiFi reads (PacBio) and mapped against GRCh38.

Ethics declaration

Procedures adhered to the tenets of the Declaration of Helsinki and were approved by the Ethics Board of the University of Campania 'Luigi Vanvitelli' and by the local ethics committee of the Radboudumc. Informed consent to genetic testing and data sharing was obtained from the subjects or their parents/legal guardians for minors.

Results

Targeted lrcDNA-seq successfully characterizes splice defects underlying IRDs using blood-derived RNA

To investigate the potential of the targeted lrcDNA-seq approach to functionally assess splice defects, we selected five probands harboring known splice-altering variants in four different IRD-associated genes that were previously published (validation cohort, Table S1).

P-1 harbors a heterozygous non-canonical splice site variant in *HGSNAT* (NM_152419.3:c.493 + 5G>A, [MIM: 610453, http://www.omim.org]) and was previously reported in this individual as likely pathogenic by Fadaie et al.[17] We generated an amplicon spanning the coding region of *HGSNAT* (exons 2–18) resulting in a 1,666-bp wild-type (WT) fragment. After sequencing, obtained reads were evaluated using the Sashimi plot feature of IGV. This revealed the skipping of exon 4 in 14.9% of the *HGSNAT* transcripts from P-1 (Figure 1A), confirming the previously published results that were obtained using a midigene assay.[17] As no variants were present within the amplicon that allowed phasing of alleles, it was not possible to conclude whether the variant causes a full splice defect (complete exon skipping) or partial defect.

Next, we investigated two Leber congenital amaurosis (LCA [MIM: 611755]) probands (P-2 and P-3) with biallelic variants in *CEP290* (MIM: 610142). Using short-read genome sequencing, Zeuli et al. previously identified a deep-intronic *CEP290* variant (NM_025114.4:c.6136-436A>G) in both individuals in *trans* with a heterozygous *CEP290* frameshift variant (P-2:c.6869del; P-3:c.6604del), demonstrated to cause a 97-nt pseudoexon inclusion.[2] Due to the large size of the complete *CEP290* transcript (7,824 bp), we assessed the subjects' RNA using primers encompassing exon 34–52 of *CEP290*. Long-read sequencing data confirmed previous results (Figure 1B). In all the reads encompassing the pseudoexon, the underlying pathogenic deep-intronic variant was present.

Next, we analyzed P-4 harboring a complex *IMPG2* allele (NM_016247.3:c.[3023-15T>A; 3023G>A] [MIM: 607056]), previously described by Vázquez-Domínguez et al.,[18] which causes a complex splice defect (p.[Gly1008-Valfs*17,Gly1008Asp,Asp1009Asnfs*14]) revealed using a midigene assay (Figure S1). We designed primers annealing to exon 13 and exon 19 of *IMPG2*, resulting in a 1,690-bp amplicon and proceeded with targeted lrcDNA-seq. Interestingly, we were able to reproduce the previous results, but also identified additional transcript isoforms that were not observed using the midigene assay (isoforms 5–7, Figure 2A). Using our lrcDNA-seq pipeline, natural exon skipping events could be observed in both conditions (WT and mutant), normal splicing (31.4% and 39.9%, respectively), exon 16 skipping (isoform 1, 18.2% and 13.3%), and exon 16 and 17 co-skipping (isoform 2, 19.5% and 16.3%). In addition, mutant-specific isoforms (isoforms 3, 4, 6, and 7) were revealed, which included the truncation of exon 15 and/or different exon skipping events (Figure 2A).

Finally, we selected a person from a previously studied family, who was diagnosed with choroideremia (P-5 [MIM: 303100]) and who harbors a complex structural variant in *CHM* (NM_00390.3: c.1510 + 693_1510 + 694ins1414−1244_1510 + 402inv [MIM: 300390]) that causes the skipping of exon 12.[16,19] We utilized the same RNA samples studied by Fadaie et al. from P-5 (male), as well as from a female family member, a hemizygous carrier of the variant.[16] The complete *CHM* transcript was amplified and subjected to analysis to verify whether a full-length approach could reveal additional undetected events. The obtained Sashimi plots for each individual

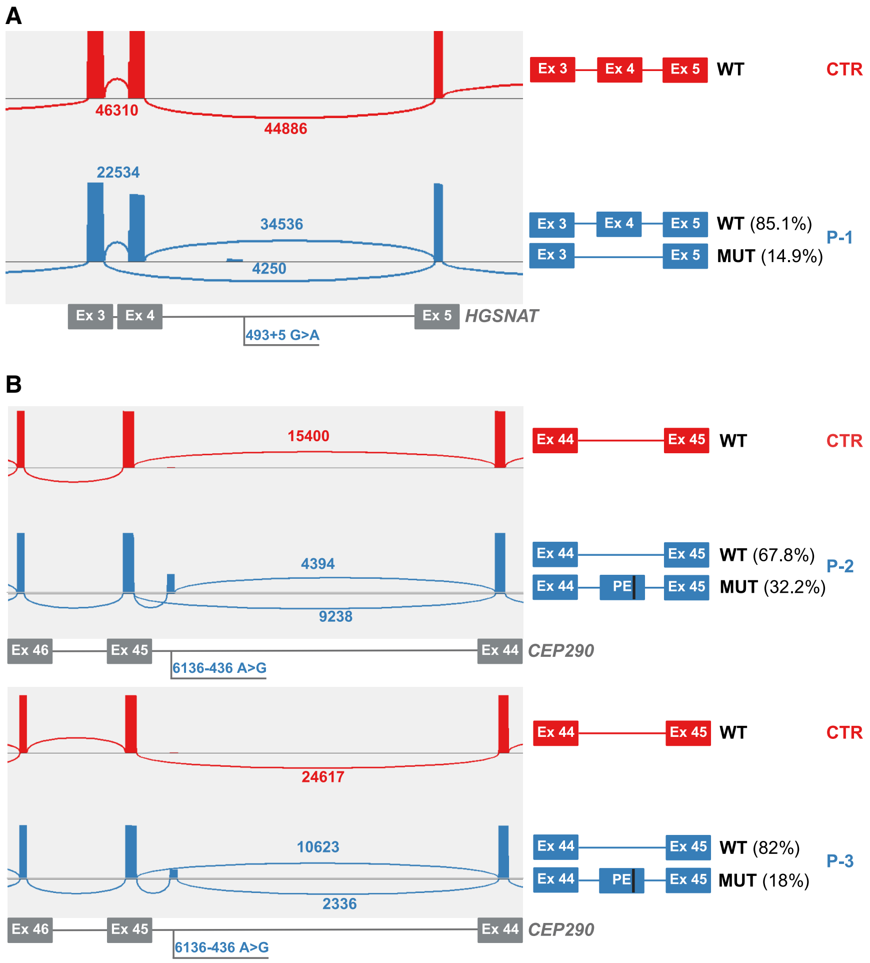

Figure 1. Targeted lrcDNA sequencing confirms splicing defects in *HGSNAT* and *CEP290*

(A) Left: Sashimi plot illustrating an enlarged view (exons 3–5) of the sequencing reads aligned to the *HGSNAT* transcript (NM_152419.3) that were obtained using control-derived cDNA (CTR, in red) and cDNA derived from subject P-1 harboring the c.493+5G>A variant (P-1, in blue). The Sashimi plots were obtained using a junction coverage min parameter of 1,000 reads. Right: Schematic illustration of the different read types observed.

(B) Left: Sashimi plot providing an enlarged view (exons 44–46) of the sequencing reads aligned to the *CEP290* transcript (NM_025114.4) obtained using control-derived cDNA (CTR, in red), and P-2- and P-3-derived cDNAs with the c.6136-436A>G variant (in blue). The Sashimi plots were obtained using a junction coverage minimum parameter of 1,000 reads. Right: Schematic illustration of the different read types observed. The numbers of reads covering the indicated exon-exon junctions are indicated in the Sashimi plots and were used to calculate the percentages provided. CTR, control; Ex, exon; MUT, mutant; P, proband; PE, pseudoexon; WT, wild-type.

are displayed in Figure 2B. Our results were in line with the previously performed assays, but surprisingly, a missed variant effect was observed in both the carrier (1.3%) and P-5 (9.8%), showing exon 11 and 12 co-skipping resulting in a frameshift (p.[(Gln451Phefs*10)]). Unfortunately, phasing of the different alleles was not possible due to the absence of variants within the amplicon.

Based on the results obtained analyzing the validation cohort, we concluded that the targeted lrcDNA-seq pipeline could be an efficient tool to detect reported transcript abnormalities and to potentially uncover missed transcripts underlying IRDs in blood-derived RNA samples.

Targeted lrcDNA-seq reveals novel splice defects in genetically unexplained IRD individuals

To assess the diagnostic utility of targeted lrcDNA-seq, we investigated five genetically unexplained IRD probands exhibiting an LCA phenotype (discovery cohort [MIM: 608553]). P-6 and P-7 harbor biallelic variants in the *NMNAT1* gene ([MIM: 608700]), one of which was classified as a variant of uncertain significance (VUS), as the potential associated splice defect remains unexplored. P-8, P-9, and P-10 have a single heterozygous pathogenic defect, and the second allele remains unobserved despite extensive genetic testing (Table S2).

P-6 was previously analyzed using exome and short-read RNA sequencing. Two in *trans* candidate variants were identified in the gene *NMNAT1*: a missense variant (NM_022787.4: c.769G>A) ACMG classified as pathogenic, and a c.-57+1del, which is classified as VUS in intron 1. SpliceAI predictions for the c.-57+1del variant include the deletion of the canonical donor site in intron 1 (delta score: 0.93) and the creation of a new donor site at the −1 position (delta score: 0.55) (Figure 3A). The relevance of this prediction is difficult to interpret as the c.-57+1del variant is located in the 5′ UTR of the gene. A recent study highlighted the importance of similar variants in *NMNAT1*, as the 5′ UTR acts as a promoter region and variants could therefore impact on transcription regulation.[20] Similarly, we speculate that the c.-57+1del variant could alter promoter efficacy, thereby affecting *NMNAT1* transcription and stability. Previously performed short-read RNA sequencing for P-6 only detected the presence of the c.769G>A allele, suggesting a strong deleterious effect of the 5′ UTR variant on transcript stability. To exclude insensitivity of the short-read sequencing, the same RNA, extracted from whole blood, was subjected to the lrcDNA-seq

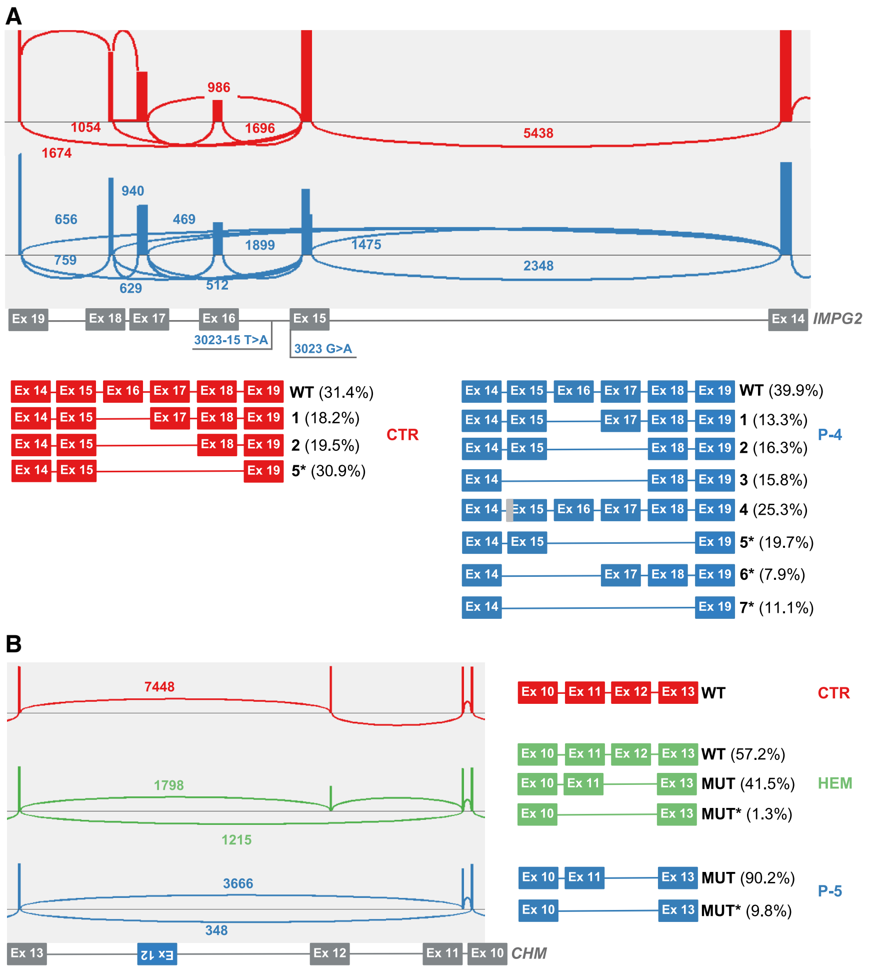

Figure 2. Targeted lrcDNA sequencing reveals novel splicing defects when studying *IMPG2* and *CHM* variants

Novel *IMPG2* and *CHM* splice isoforms were identified that were not previously detected using a midigene-based splice assay[18] or RNA-seq studies.[16,19]

(A) Top: Sashimi plot providing an enlarged view (exons 14–19) of the sequencing reads aligned to the *IMPG2* transcript (NM_016247.3) obtained using control-derived cDNA (CTR, in red) and subject-derived cDNA (P-4, in blue). The numbers of reads covering the indicated exon-exon junctions are indicated. The Sashimi plots were obtained using a junction coverage minimum parameter of 100 reads. Bottom: Schematic representation of the different *IMPG2* isoforms that were identified.

(B) Left: Sashimi plot providing an enlarged view (exons 10–13) of the sequencing reads aligned to the *CHM* transcript (NM_00390.3) obtained using control-derived cDNA (CTR, in red), cDNA derived from a hemizygous carrier (HEM, in green), and subject-derived cDNA (P-5, in blue). The numbers of reads covering the indicated exon-exon junctions are indicated. The Sashimi plots were obtained using a junction coverage minimum parameter of 100 reads. Right: Schematic representation of the identified transcripts in the different samples. The numbers of reads covering the indicated exon-exon junctions are indicated in the Sashimi plots and were used to calculate the percentages provided. *Read types identified using the targeted lrcDNA-seq pipeline that were not detected in previously performed studies.

pipeline. The full *NMNAT1* transcript was amplified, and IGV visualization of the obtained long-reads confirmed the previous observations as only the c.769G>A allele could be observed. This suggests a nearly complete degradation of the *NMNAT1* transcript encompassing the c.-57+1del variant (Figure 3A).

P-7 harbors the c.769G>A *NMNAT1* variant in *trans* with the c.-57G>A substitution classified as a VUS. SpliceAI predictions suggested the loss of the canonical donor site (delta score: 0.47) in intron 1 and the gain of a cryptic donor site (delta score: 0.25) 122 bp downstream, leading to an exon 1 extension (Figure 3B). The lrcDNA-seq reads indicated that the majority of sequencing reads harbored the missense c.769G>A variant, with a small proportion of the reads (0.15%) containing a "G" (Figure 3B).

Based on these results, we collected functional evidence at the RNA level supporting the pathogenicity of these 5′ UTR variants, which enabled their reclassification as pathogenic following the ACMG-AMP variant classification guidelines, hence considering these individuals genetically explained (Table 1).

Detection of a mobile element insertion as a potential founder variant in monoallelic Italian LCA subjects

Next, we investigated three monoallelic *NMNAT1* probands (P-8, P-9, and P-10, Table S2). For all, whole-blood RNA was obtained and subjected to the lrcDNA-seq pipeline to amplify the entire *NMNAT1* transcript. Gel electrophoresis analysis of the obtained PCR products revealed fragments of unexpected sizes, suggesting the presence of longer, alternatively spliced products (Figure S2). Analysis of the reads in IGV revealed an enrichment of transcripts bearing the previously identified missense variants (allele 1), with an average percentage of 91% for all three individuals analyzed (Figures 4A and 4B). The remaining reads could be divided into two types: normally spliced reads and reads containing an *NMNAT1* exon 2 elongation, followed by an insertion of 875 nt (Figures 4A–4C). The same insertion was observed in all three monoallelic subjects analyzed, with 99.5% identity to a part of an SVA_F retrotransposon element, originating from chr6:122849419-122850290 (GRCh38). To elucidate this transcriptional alteration at the genomic level, we

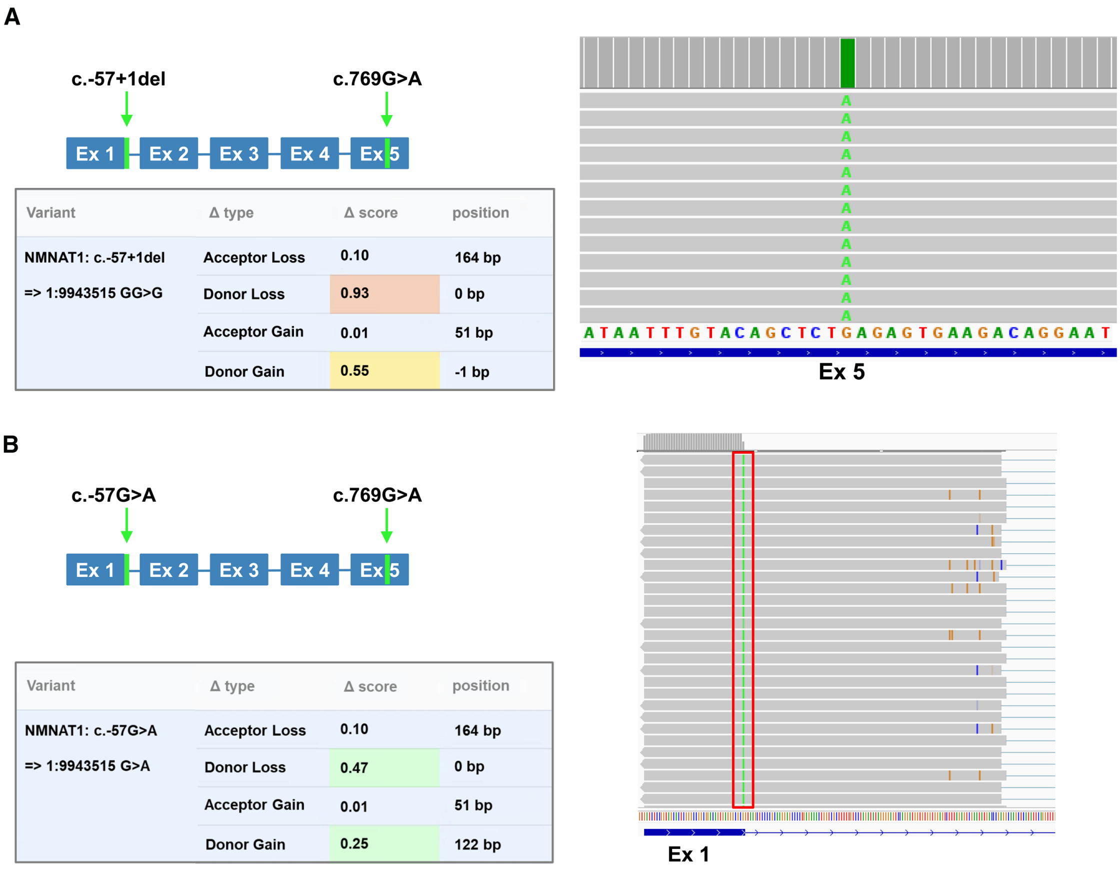

Figure 3. Targeted lrcDNA sequencing reveals deleterious effects of 5′ UTR variants in *NMNAT1*

(A) Left: Schematic representation of the previously identified variants that were found in *NMNAT1* (NM_022787.4) in P-6 (allele 1: c.769G>A, allele 2: c.-57+1del). The table below provides the SpliceAI prediction scores (range 0–1) of the 5′ UTR variant present on allele 2. Based on these scores, the variant is predicted to cause the loss of a donor splice site at position 0, together with the gain of a strong donor site at position −1; this could lead to transcript instability. Right: IGV visualization providing an enlarged view (exon 5) of the sequencing reads aligned to the *NMNAT1* transcript (NM_022787.4, 10,784 total reads) that were obtained using subject-derived cDNA (P-6). The green line represents the c.769G>A variant. No sequencing reads without this variant were observed, suggesting that allele 2 is completely degraded possibly due to altered stability.

(B) Left: Schematic representation of the variants that were found in P-7 in the *NMNAT1* gene (allele 1: c.769G>A, allele 2: c.-57G>A). The table below provides the SpliceAI prediction scores (range 0–1) for the 5′ UTR variant present on allele 2. Based on these scores, the variant is predicted to cause the loss of a donor splice site in position 0, associated with the gain of another donor site in position 122. Based on this prediction, a 122-bp elongation of exon 1 should be observed. Right: IGV visualization providing an enlarged view (exon 1) of part of the sequencing reads aligned to the *NMNAT1* transcript (172,847 total reads) obtained using subject-derived cDNA (P-7). Only 0.15% of the total reads harbored the 5′ UTR variant (red box) and were derived from allele 2, suggesting an almost complete transcript degradation possibly due to altered stability.

performed long-read genome sequencing on DNA of P-9. Interestingly, this analysis revealed the insertion of the complete SVA_F retrotransposon sequence (2,691 bp) in intron 2 of *NMNAT1* (Figures 4D and 4E): NC_000001.11: g.9972228_9972229ins[G; NC_000006.12:g.122847777_122850290inv; A38; GACTAGAGAACC].

The genome sequencing data, along with the targeted lrcDNA-seq reads, suggest the creation of a new splice donor site at position 875 bp of the inserted SVA_F element (914 bp distal from the exon 2-intron 2 boundary) which competes with the canonical donor site, ultimately resulting in the complex event observed at the RNA level and leading to the introduction of a premature stop codon (p.[Tyr41Ser*57,=]). To support this hypothesis, a sequence of 200 bp from the inserted SVA_F element surrounding the possible newly created donor site was analyzed using SPLICEATOR (version 2.1).[21] Among the possible splice sites highlighted, the donor site present in position 875 had a reliability score of 87%, which supports our hypothesis. Since ∼50% of the reads derived from the SVA_F allele show WT splicing, the variant only causes a partial splice defect effect (Figure 4C). Based on these results, the variant was ACMG classified as pathogenic[13] (Table 1).

Since the *NMNAT1* insertion was identified in three unrelated Italian LCA probands, we screened 11 additional monoallelic *NMNAT1* subjects of various origins using variant-specific breakpoint primers. As a result, we identified one other Italian LCA-affected individual harboring the SVA_F retrotransposon insertion. This suggests that this insertion is a potential founder variant in the Italian population.

Table 1. Overview of pathogenic variants identified in this study

Study ID	Variant details		ACMG classification
	DNA	Protein	
P-6	c.-57+1del	p.0?	pathogenic
P-7	c.-57G>A	p.0?	pathogenic
P-8	SVA_F ins	p.[Tyr41Ser*57,=]	pathogenic
P-9	SVA_F ins	p.[Tyr41Ser*57,=]	pathogenic
P-10	SVA_F ins	p.[Tyr41Ser*57,=]	pathogenic

NC_000001.11:g.9972228_9972229ins[G;NC_000006.12:g.122847777_122850290inv;A[38];GACTAGAGAACC]. ACMG, variant classification according to the ACMG-AMP guidelines; P, proband; Study ID, subject identification as used in the present study; SVA_F ins, mobile element insertion.

Targeted lrcDNA-seq for IRD transcripts with low expression in blood

A limitation of the targeted lrcDNA-seq approach is represented by the low expression of a significant number of IRD transcripts in blood-derived samples. According to GTEx data (GTEx Portal, https://www.gtexportal.org), of the 282 IRD genes expressed in blood, 155 have a transcripts per million (TPM) expression value higher than 0.5 (expression cutoff), thus being suitable for the lrcDNA-seq pipeline. However, to further assess the extent of the pipeline coverage, we selected additional RNAs extracted from lymphoblastoid cell lines of three subjects with variants in *ABCA4* (MIM: 601691), *CRB1* (MIM: 604210), and *RPGRIP1* (MIM: 605446) (TPM values <0.1). The coverage for these transcripts was observed only when using retina-derived RNA (positive control sample), addressing the importance of blood gene expression for a successful analysis to be performed (data not shown). This was in contrast to our *IMPG2* findings showing sufficient coverage despite a TPM value of 0.06 (Figure 2A). In this case, a smaller amplicon spanning only exons 13–19 of the transcript was amplified, which possibly explains why this limitation could be resolved.

Discussion

In this study, we investigated the diagnostic value of a targeted lrcDNA-seq method applied to blood-derived RNA to validate and identify novel splice-affecting variants underlying IRDs. Through the analysis of the validation cohort, we were able to detect the previously observed findings. Additionally, in *IMPG2* or *CHM* we identified previously undetected and low-abundant transcript alterations. These findings highlighted (1) the higher sensitivity of lrcDNA-seq compared to midigene-based splice assays and short-read RNA-seq, (2) the added value of amplifying the entire (or longer unbiased portions of) transcript and of studying a variant in a broader natural genomic context, and (3) the phasing: amplification of the entire transcripts has the potential to discriminate different alleles' transcripts. Nevertheless, the possibility of phasing depends on the presence of allele-discriminating SNPs or variants within the mRNA/cDNA amplicon. Although the targeted nature of our method (requiring a PCR amplification step) introduces a bias and limits the detection of novel fusion transcripts and UTRs, it also holds several advantages, including higher throughput, lower costs, and deeper sequencing of the transcript of interest. These features make it an attractive approach for potential implementation in a diagnostic setting, specifically when compared to more expensive transcriptome-wide approaches.

Next, we proceeded with the characterization of five genetically unexplained LCA subjects, with variants identified in the *NMNAT1* gene. In P-6 and P-7, two previously identified VUSs were successfully validated and reclassified as pathogenic. In both individuals, the deleterious effect was not caused by a splicing defect, but we hypothesize that the variants located in the 5′ UTR led to transcripts' instability and hence degradation through NMD. Overall, these results highlight not only the application of the targeted lrcDNA-seq approach to investigate the pathogenicity of regulatory variants but also the relevance of the deep coverage obtained with long-read platforms able to detect low abundant transcript isoforms. The latter case was demonstrated in P-7 RNA, where we were able to detect even 0.15% of reads bearing the c.-57G>A *NMNAT1* variant, suggesting that most of these transcripts are degraded.

In the analyzed LCA-affected subjects P-8, P-9, and P-10, we were able to identify a deleterious insertion of an SVA_F retrotransposon in intron two of *NMNAT1*, which was not detected using short-read genome sequencing in the past due to its sequence complexity. Mobile elements can act as mutagens with an estimated insertional event frequency of 1/20 live births and with more than 120 events already associated with human diseases.[22] However, it should be noted that detection of mobile elements is hampered by (1) the limits of the short-read approaches unable to cover long, repetitive regions, (2) the poor implementation of specific computational methods in routine diagnostics able to

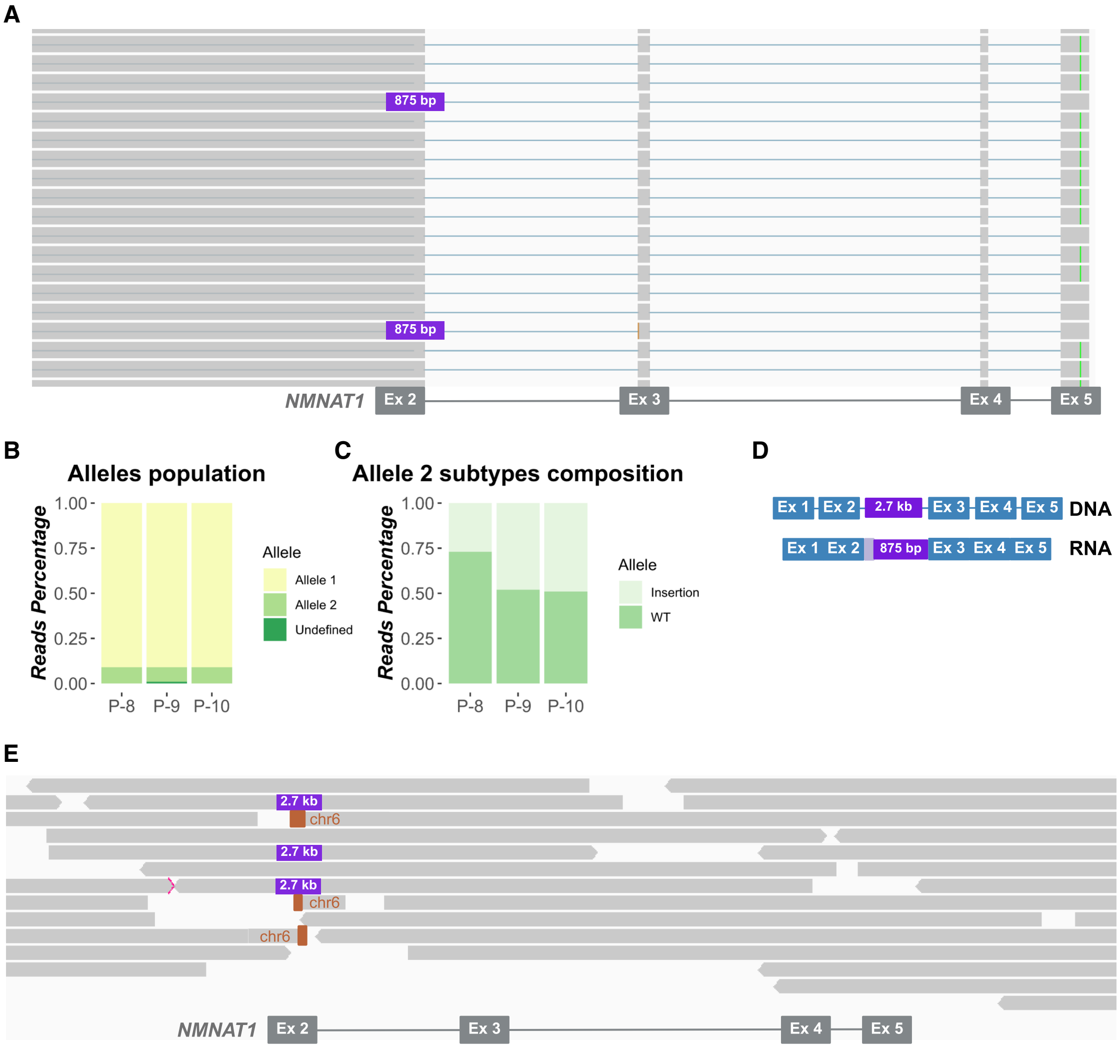

Figure 4. Targeted lrcDNA sequencing and long-read genome sequencing identify and define the boundaries of SVA_F insertion in *NMNAT1*

(A) IGV image showing an enlarged view (exons 2–5) of the sequenced reads obtained for the *NMNAT1* transcript (NM_022787.4, 147,915 total reads) using subject-derived cDNA (P-9). Three allele subpopulations can be recognized: (1) harboring the c.769G>A variant (allele 1, highlighted in green), (2) WT reads without the c.769G>A variant, and (3) reads containing an 875-bp insertion (allele 2, indicated with a purple square). A BLAST analysis revealed that the inserted 875 bp constituted a partial SVA_F mobile element. A similar 875-bp insertion event was observed in lrcDNA sequencing reads of P-8 and P-10 as well (data not shown), in *trans* with a previously reported pathogenic *NMNAT1* variant.

(B) Relative estimated percentages of the two different alleles that are present in each sample, as calculated using the targeted lrcDNA sequencing data. Allele 1: c.769G>A (P-8 and P-9), c.634G>A (P-10); allele 2: mixed population of WT reads and reads harboring the SVA_F element insertion; Undefined: reads harboring a C or a T in c.769 position probably due to sequencing artifacts; therefore, these are not included in the calculations.

(C) Estimated percentages of the two different subpopulations that were observed for transcripts derived from allele 2, showing either WT splicing or aberrant splicing.

(D) To resolve the SVA_F insertion event at the genomic level, long-read genome sequencing was performed. Schematic representation of the genomic DNA and cDNA sequencing results; at the genomic level, the complete retrotransposon element (2,691 bp) was found to be inserted. At the RNA level, an elongation of exon 2 and partial inclusion of the SVA_F element was revealed (875 bp).

(E) IGV image providing an enlarged view (exon 2–5) of the genome sequencing reads mapped against the *NMNAT1* gene (14 total reads) derived from subject-derived genomic DNA (P-9). The purple box indicates the 2,691-bp SVA_F element insertion in intron 2 of *NMNAT1*. The sequencing reads highlighted in orange map to chromosome 6, highlighting the genomic origin of the SVA_F element.

recognize these events, and (3) the low number of pathogenic insertional events described so far.[23] This finding highlights the ability and added value of lrcDNA-seq to detect novel mutational events that could have been missed using short-read approaches and its potential to resolve part of the missing heritability that is reported for IRDs.

Other studies have been performed to estimate the diagnostic relevance of RNA-based approaches in IRDs. Weisschuh et al. coupled the RNA-seq from whole-blood

samples with genome sequencing to support variant classification when a transcript defect was predicted to be involved in the disease pathogenesis. However, most of the variants could not be evaluated for the low blood expression of the related transcripts. Moreover, the authors showed the impossibility of detecting some transcript alterations (e.g., frameshift) due to the low coverage caused by the activation of NMD and/or the short reads lengths.[24] Although not investigated in the present study, this limitation caused by NMD could be overcome by the use of RNA derived from lymphoblastoid cell lines, which can be treated with NMD inhibitors (e.g., cycloheximide), to allow further characterization of the magnitude of specific variants. Nevertheless, we have shown that by employing a deep-sequencing approach such as our lrcDNA-seq method, it is possible to pick up extremely low abundant transcripts that are possibly subjected to NMD such as the allele derived from the NM_00390.3: c.1510 + 693_1510 + 694ins1414−1244_1510 + 402inv variant in the carrier, P-5's sibling (1.3% of the sequencing reads).

Another example study combined the midigene assays or RT-PCR-based approach with nanopore sequencing to functionally characterize predicted splice-altering variants in individuals affected by IRDs.[25] Despite being successful in detecting aberrant transcripts, doubts about the effectiveness of these approaches have been raised when applied to tissues not directly associated with the disease pathogenesis. The existence of cell-specific transcript isoforms and splicing is a well-known phenomenon, and this limitation could also be true for the targeted lrcDNA method.[26] Further efforts are required to validate the technique on more biologically relevant samples (e.g., photoreceptor precursor cells).

Based on our results, we conclude that the proposed targeted lrcDNA-seq tool can successfully be performed on easily accessible tissues such as blood to contribute to genetic diagnostics for IRDs when performed in parallel with genomic screening. Considering the relatively small sample number analyzed in the discovery cohort, follow-up studies are required to optimize the technique to investigate its full potential and its translation to other diseases and IRD-associated genes. Nevertheless, we speculate that this approach could be extended to a wide range of genetic diseases in case a strong splicing-affecting variant is identified and requires functional validation to complete a genetic diagnosis, or when a second pathogenic variant cannot be detected by first-step diagnostic analysis in the presence of a first hit in a gene strongly correlated to the individual's phenotype.

The use of RT-PCR and subsequent long-read targeted sequencing directly performed on blood-derived cDNA makes this pipeline a faster and cheaper alternative compared to transcriptome-wide approaches (e.g., Iso-Seq or short-read RNA-seq) and more reliable when compared to artificial systems (e.g., midigene assays). Therefore, this method could aid in providing more affected individuals with genetic diagnoses and potentially improve diagnostics rates, ultimately allowing more effective genetic counseling and better guidance toward gene-specific therapies in the future.

Data and code availability

All the data generated during the present study can be obtained from the corresponding author upon reasonable request. Identified genetic variants have been reposited at the Leiden Open Variation Database (www.lovd.nl/NMNAT1).

Acknowledgments

The authors would like to acknowledge the valuable contributions and technical support of Saskia D. van der Velde-Visser, Lara Holtes, Kim Rodenburg, Erica Boonen, Zelia Corradi, and Irene Vázquez-Domínguez. The TPM values described in this paper were obtained from the GTEx Portal and dbGaP accession number phs000424.v8.p2 on September 3, 2024. Illustrations were created with BioRender.com. This work was financially supported by the Landelijke Stichting voor Blinden en Slechtzienden, Ooglijders, Stichting Blindenhulp, Stichting Oogfonds Nederland, Verbetering van het Lot der Blinden (to F.P.M.C., S.R., D.M.P., and S.E.d.B.), the Italian Ministry of Research (MUR) under the EJP RD program (project Solve-RET to S.B.), the Fondazione Telethon for grants TGM23MFU01 and TGM22GM03 (to S.B.), and the Italian Ministry of Health (Ricerca Corrente and Ricerca Finalizzata RF-2019-12369368 to S.S. and E.M.V.).

Author contributions

D.C.: conceptualization, methodology, investigation, visualization, and writing – original draft. R.Z.: conceptualization. G.A.: conceptualization, methodology, and resources; M.K.: resources. R.T.: software and formal analysis. K.A.R.-C.: resources. M.K.: investigation and data curation. F.S.: resources. S.S.: resources. E.M.V.: resources. F.P.M.C.: conceptualization and supervision. S.B.: conceptualization, supervision, and writing – review & editing. S.R.: conceptualization, supervision, and writing – review & editing. D.M.P.: conceptualization, supervision, writing – review & editing, project administration, and funding acquisition. S.E.d.B.: conceptualization, supervision, writing – review & editing, project administration, and funding acquisition.

Declaration of interests

The authors declare no competing interests.

Supplemental information

Supplemental information can be found online at https://doi.org/10.1016/j.xhgg.2025.100442.

Web resources

Franklin Genoox platform: https://franklin.genoox.com/.
GTEx Portal: https://www.gtexportal.org.
OMIM: http://www.omim.org.
RetNet: http://sph.uth.edu/retnet.

Received: January 9, 2025
Accepted: April 15, 2025

References

1. Karali, M., Testa, F., Di Iorio, V., Torella, A., Zeuli, R., Scarpato, M., Romano, F., Onore, M.E., Pizzo, M., Melillo, P., et al. (2022). Genetic epidemiology of inherited retinal diseases in a large patient cohort followed at a single center in Italy. Sci. Rep. *12*, 20815.
2. Zeuli, R., Karali, M., de Bruijn, S.E., Rodenburg, K., Scarpato, M., Capasso, D., Astuti, G.D.N., Gilissen, C., Rodríguez-Hidalgo, M., Ruiz-Ederra, J., et al. (2024). Whole genome sequencing identifies elusive variants in genetically unsolved Italian inherited retinal disease patients. HGG Adv. *5*, 100314.
3. Sundaresan, Y., Banin, E., and Sharon, D. (2023). Exonic Variants that Affect Splicing - An Opportunity for "Hidden" Mutations Causing Inherited Retinal Diseases. Adv. Exp. Med. Biol. *1415*, 183–187.
4. Anna, A., and Monika, G. (2018). Splicing mutations in human genetic disorders: examples, detection, and confirmation. J. Appl. Genet. *59*, 253–268.
5. Kapustin, Y., Chan, E., Sarkar, R., Wong, F., Vorechovsky, I., Winston, R.M., Tatusova, T., and Dibb, N.J. (2011). Cryptic splice sites and split genes. Nucleic Acids Res. *39*, 5837–5844.
6. Jaganathan, K., Kyriazopoulou Panagiotopoulou, S., McRae, J.F., Darbandi, S.F., Knowles, D., Li, Y.I., Kosmicki, J.A., Arbelaez, J., Cui, W., Schwartz, G.B., et al. (2019). Predicting Splicing from Primary Sequence with Deep Learning. Cell *176*, 535–548.e24.
7. Qian, X., Wang, J., Wang, M., Igelman, A.D., Jones, K.D., Li, Y., Wang, K., Goetz, K.E., Birch, D.G., Yang, P., et al. (2021). Identification of Deep-Intronic Splice Mutations in a Large Cohort of Patients With Inherited Retinal Diseases. Front. Genet. *12*, 647400.
8. Khan, M., Cornelis, S.S., Pozo-Valero, M.D., Whelan, L., Runhart, E.H., Mishra, K., Bults, F., AlSwaiti, Y., AlTalbishi, A., De Baere, E., et al. (2020). Resolving the dark matter of ABCA4 for 1054 Stargardt disease probands through integrated genomics and transcriptomics. Genet. Med. *22*, 1235–1246.
9. Gaildrat, P., Killian, A., Martins, A., Tournier, I., Frébourg, T., and Tosi, M. (2010). Use of splicing reporter minigene assay to evaluate the effect on splicing of unclassified genetic variants. Methods Mol. Biol. *653*, 249–257.
10. Wilkie, S.E., Vaclavik, V., Wu, H., Bujakowska, K., Chakarova, C.F., Bhattacharya, S.S., Warren, M.J., and Hunt, D.M. (2008). Disease mechanism for retinitis pigmentosa (RP11) caused by missense mutations in the splicing factor gene. Mol. Vis. *14*, 683–690.
11. Jurkute, N., Cancellieri, F., Pohl, L., Li, C.H.Z., Heaton, R.A., Reurink, J., Bellingham, J., Quinodoz, M., Yioti, G., Stefaniotou, M., et al. (2022). Biallelic variants in coenzyme Q10 biosynthesis pathway genes cause a retinitis pigmentosa phenotype. NPJ Genom. Med. *7*, 60.
12. Chandrasekhar, S., Lin, S., Jurkute, N., Oprych, K., Estramiana Elorrieta, L., Schiff, E., Malka, S., Wright, G., Michaelides, M., Mahroo, O.A., et al. (2024). Investigating Splice Defects in USH2A Using Targeted Long-Read Sequencing. Cells *13*, 1261.
13. Richards, S., Aziz, N., Bale, S., Bick, D., Das, S., Gastier-Foster, J., Grody, W.W., Hegde, M., Lyon, E., Spector, E., et al. (2015). Standards and guidelines for the interpretation of sequence variants: a joint consensus recommendation of the American College of Medical Genetics and Genomics and the Association for Molecular Pathology. Genet. Med. *17*, 405–424.
14. Kibbe, W.A. (2007). OligoCalc: an online oligonucleotide properties calculator. Nucleic Acids Res. *35*, W43–W46.
15. Thorvaldsdottir, H., Robinson, J.T., and Mesirov, J.P. (2013). Integrative Genomics Viewer (IGV): high-performance genomics data visualization and exploration. Brief Bioinform *14*, 178–192.
16. Fadaie, Z., Neveling, K., Mantere, T., Derks, R., Haer-Wigman, L., den Ouden, A., Kwint, M., O'Gorman, L., Valkenburg, D., Hoyng, C.B., et al. (2021). Long-read technologies identify a hidden inverted duplication in a family with choroideremia. Hum. Genet. Genom. Adv. *2*, 100046.
17. Fadaie, Z., Whelan, L., Ben-Yosef, T., Dockery, A., Corradi, Z., Gilissen, C., Haer-Wigman, L., Corominas, J., Astuti, G.D.N., de Rooij, L., et al. (2021). Whole genome sequencing and *in vitro* splice assays reveal genetic causes for inherited retinal diseases. NPJ Genom. Med. *6*, 97.
18. Vázquez-Domínguez, I., Li, C.H.Z., Fadaie, Z., Haer-Wigman, L., Cremers, F.P.M., Garanto, A., Hoyng, C.B., and Roosing, S. (2022). Identification of a Complex Allele in IMPG2 as a Cause of Adult-Onset Vitelliform Macular Dystrophy. Invest Ophth Vis Sci. *63*, 27.
19. van den Hurk, J.A.J.M., van de Pol, D.J.R., Wissinger, B., van Driel, M.A., Hoefsloot, L.H., de Wijs, I.J., van den Born, L.I., Heckenlively, J.R., Brunner, H.G., Zrenner, E., et al. (2003). Novel types of mutation in the choroideremia (CHM) gene: a full-length L1 insertion and an intronic mutation activating a cryptic exon. Hum. Genet. *113*, 268–275.
20. Coppieters, F., Todeschini, A.L., Fujimaki, T., Baert, A., De Bruyne, M., Van Cauwenbergh, C., Verdin, H., Bauwens, M., Ongenaert, M., Kondo, M., et al. (2015). Hidden Genetic Variation in LCA9-Associated Congenital Blindness Explained by 5'UTR Mutations and Copy-Number Variations of NMNAT1. Hum. Mutat. *36*, 1188–1196.
21. Scalzitti, N., Kress, A., Orhand, R., Weber, T., Moulinier, L., Jeannin-Girardon, A., Collet, P., Poch, O., and Thompson, J.D. (2021). Spliceator: multi-species splice site prediction using convolutional neural networks. BMC Bioinf. *22*, 561.
22. Wijngaard, R., Demidov, G., O'Gorman, L., Corominas-Galbany, J., Yaldiz, B., Steyaert, W., de Boer, E., Vissers, L.E.L. M., Kamsteeg, E.J., Pfundt, R., et al. (2024). Mobile element insertions in rare diseases: a comparative benchmark and reanalysis of 60,000 exome samples. Eur. J. Hum. Genet. *32*, 200–208.
23. Torene, R.I., Galens, K., Liu, S., Arvai, K., Borroto, C., Scuffins, J., Zhang, Z., Friedman, B., Sroka, H., Heeley, J., et al. (2020). Mobile element insertion detection in 89,874 clinical exomes. Genet. Med. *22*, 974–978.
24. Weisschuh, N., Mazzola, P., Zuleger, T., Schaeferhoff, K., Kühlewein, L., Kortüm, F., Witt, D., Liebmann, A., Falb, R., Pohl, L., et al. (2024). Diagnostic genome sequencing improves diagnostic yield: a prospective single-centre study in 1000 patients with inherited eye diseases. J. Med. Genet. *61*, 186–195.

25. Maggi, J., Feil, S., Gloggnitzer, J., Maggi, K., Bachmann-Gagescu, R., Gerth-Kahlert, C., Koller, S., and Berger, W. (2024). Nanopore Deep Sequencing as a Tool to Characterize and Quantify Aberrant Splicing Caused by Variants in Inherited Retinal Dystrophy Genes. Int. J. Mol. Sci. *25*, 9569.

26. Wojcik, M.H., Reuter, C.M., Marwaha, S., Mahmoud, M., Duyzend, M.H., Barseghyan, H., Yuan, B., Boone, P.M., Groopman, E.E., Délot, E.C., et al. (2023). Beyond the exome: What's next in diagnostic testing for Mendelian conditions. Am. J. Hum. Genet. *110*, 1229–1248.

HGGA, Volume *6*

Supplemental information

Targeted long-read cDNA sequencing reveals novel splice-altering pathogenic variants causing retinal dystrophies

Dalila Capasso, Roberta Zeuli, Gavin Arno, Michael Kwint, Raoul Timmermans, Karla A. Ruiz-Ceja, Marianthi Karali, Francesca Simonelli, Sabrina Signorini, Enza Maria Valente, Frans P.M. Cremers, Sandro Banfi, Susanne Roosing, Daan M. Panneman, and Suzanne E. de Bruijn

TABLE OF CONTENTS

SUPPLEMENTAL FIGURES………2

Figure S1……2

Figure S2……3

SUPPLEMENTAL TABLES………4

Table S1 (Large Excel file, uploaded separately)

Table S2 (Large Excel file, uploaded separately)

Table S3……4

SUPPLEMENTAL REFERENCES…………………………………………………………………………………………………5

SUPPLEMENTAL FIGURES

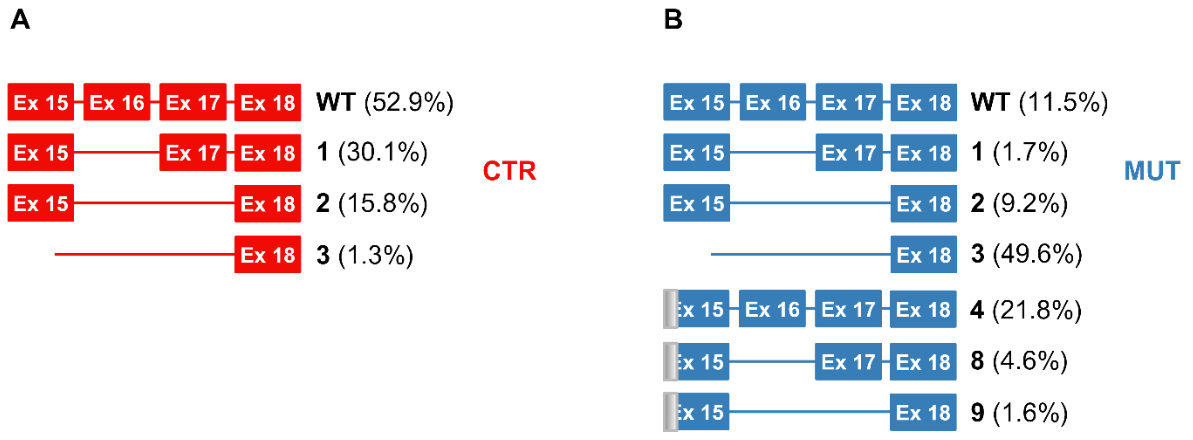

Figure S1. Schematic representation of the midigene assay results described by Vázquez-Domínguez et al. studying the *IMPG2* c.[3023-15T>A;3023G>A] variant. A midigene assay using wildtype and mutant midigene constructs containing exons 15-18 of *IMPG2* was previously performed. The different isoforms identified in these experiments are schematically illustrated. (A) Midigene results obtained using the wildtype *IMPG2* construct (CTR, in red). Besides the normally spliced *IMPG2* transcript (wildtype (WT)), also isoforms lacking exon 16 (isoform 1), exons 16 and 17 (isoform 2) and exons 15, 16 and 17 (isoform 3) were identified. (B) Midigene results obtained using the mutated *IMPG2* construct (MUT, in blue). Besides the wildtype isoform and isoforms 1-3, also mutant-specific isoforms were detected that included a truncation of exon 15 (isoform 4) with or without co-skipping of exon 16 (isoform 8) or exons 16 and 17 (isoform 9). Percentages indicate the relative abundance of each isoform as calculated using a semi-quantitative analysis of the RT-PCR results as described by Vázquez-Domínguez et al. [1]. Ex, Exon.

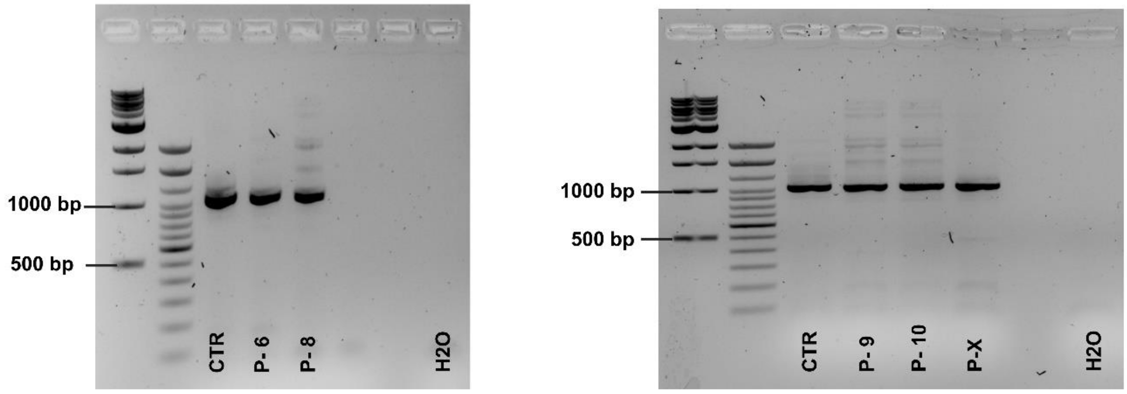

Figure S2. Targeted long-read cDNA sequencing identifies an SVA_F insertion in *NMNAT1*. Gel electrophoresis analysis of RT-PCR products. The complete *NMNAT1* transcript (exon 1 – exon 5) was amplified using P-6-, P-8-, P-9- and P-10-derived cDNA. Besides the expected wildtype product (993 bp), products of unexpected sizes were observed in all three affected monoallelic individuals. Bp, Base pairs; CTR, Control; P, Proband.

SUPPLEMENTAL TABLES

Table S3. Primer sequences and PCR conditions used.

Transcript	Primer F (5'-3')	Primer R (5'-3')	Length	PCR conditions
HGSNAT	GATGGATCAGGCTTTGCTAC	GTGGGACTGGTTGTCCTTC	1666 bp	Q5, 63C, 2 min
IMPG2	GCTGAATCTGCGTCTCTAAC	TCCATCTTCTCCAGGCTTC	1690 bp	Q5, 63C, 2 min
CHM	TCAAGATGGCGGATACTCTC	AAGTCTCCGAGTTAGCCTCTG	1920 bp	Q5, 63C, 2 min
CEP290	CTAGAAACACGGGCAACTTG	CTTTCAGCTCCACTTTGGTC	2717 bp	Q5, 64C, 2 min
NMNAT1	GATCTCCGGTAGCACTCGG	CCCAGATTGTTTCAGATCCCCA	993 bp	Q5, 68C, 2 min

Primer sequences used for RT-PCR analysis and targeted long-read cDNA sequencing. F, Forward primer sequence; R, Reverse primer sequence; Length, Size of amplified PCR product in base pairs (bp); PCR conditions, PCR conditions employed for each specific PCR reaction specifying (1) the polymerase used, (2) the annealing temperature and (3) the elongation time applied in minutes.

SUPPLEMENTAL REFERENCES

1. Vázquez-Domínguez, I., et al., *Identification of a Complex Allele in IMPG2 as a Cause of Adult-Onset Vitelliform Macular Dystrophy.* Investigative Ophthalmology & Visual Science, 2022. **63**(5).
